# Diagnostic performance of the EU TI‐RADS and ACR TI‐RADS scoring systems in predicting thyroid malignancy

**DOI:** 10.1002/edm2.434

**Published:** 2023-06-16

**Authors:** Hiba‐Allah Chatti, Ibtissem Oueslati, Aymen Azaiez, Jihen Marrakchi, Seif Boukriba, Habiba Mizouni, Slim Haouet, Ghazi Besbes, Meriem Yazidi, Melika Chihaoui

**Affiliations:** ^1^ Department of Endocrinology La Rabta university hospital, Faculty of medicine, University of Tunis‐El Manar Tunis Tunisia; ^2^ Department of Oto‐Rhino‐laryngology La Rabta university Hospital, Faculty of medicine, University of Tunis‐El Manar Tunis Tunisia; ^3^ Department of Radiology La Rabta university hospital, Faculty of medicine, University of Tunis‐El Manar Tunis Tunisia; ^4^ Department of Pathology La Rabta university hospital, Faculty of medicine, University of Tunis‐El Manar Tunis Tunisia

**Keywords:** diagnostic imaging, malignancy risk, thyroid carcinoma, thyroid nodule, ultrasound classifications

## Abstract

**Introduction:**

Several ultrasound scoring systems have been developed to stratify the risk of malignancy of thyroid nodules, including ACR (American College of Radiology) and EU (European) TI‐RADS. This study aimed to assess the diagnostic performance of these two classifications using histology as a reference standard.

**Methods:**

It was a single‐centre, retrospective study including 156 patients who underwent thyroidectomy. Ultrasound data of 198 nodules (99 malignant nodules and 99 benign nodules) were analysed. Both classifications were applied for all nodules.

**Results:**

Ultrasound criteria associated with malignancy were solid composition (OR=7.81; *p* < 10^−3^), hypoechoic character (OR=16.42; *p* < 10^−3^), irregular contours (OR=7.47; *p* < 10^−3^), taller‐than‐wide shape (OR=3.58; *p* = 0.02), microcalcifications (OR=3.02; *p* = .006) and the presence of cervical adenopathy (OR=3.89; *p* = .006). The prevalence of malignancy was 15.5%, 69% and 76.9% for EU TI‐RADS categories 3, 4 and 5, respectively. It was 33.3%, 57% and 91.1% for ACR TI‐RADS categories 3, 4 and 5, respectively. For category 5, EU TI‐RADS and ACR TI‐RADS had sensitivities of 60% and 41%, specificities of 82% and 96%, respectively. For categories 4 and 5 combined, the diagnostic performance of these two classification systems became comparable with a sensitivity of 89% and 86% for EU‐TIRADS and ACR‐TIRADS, respectively. The area under the ROC curve was 0.81 for the EU TI‐RADS classification and 0.82 for the ACR TI‐RADS classification.

**Conclusions:**

EU TI‐RADS and ACR TI‐RADS scoring systems seem to be comparable in predicting malignancy in thyroid nodules.

## Graphical abstract

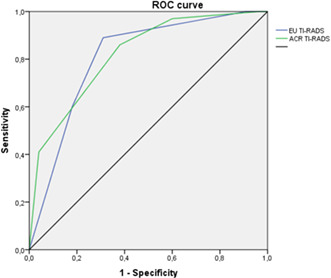

## 1 INTRODUCTION

A thyroid nodule is defined as a lesion within the thyroid gland that is radiologically distinct from the surrounding thyroid parenchyma.^1^ Since the widespread use of cervical ultrasound, thyroid nodules have become a common entity with a prevalence of 60%.^2^, ^3^ However, thyroid cancer remains a relatively rare entity accounting for less than 10% of thyroid nodules.^4^ Thyroid ultrasound and fine needle aspiration cytology represent the standard of care for evaluating thyroid nodules. The thyroid ultrasound is widely used and recognized as the first tool to characterize thyroid nodules. Several ultrasound scoring systems have been developed to estimate the risk of malignancy and to identify nodules deserving fine needle aspiration cytology. Among the most recent and recognized Thyroid Imaging Reporting and Data System (TI‐RADS) classifications, the American College of Radiology (ACR) advanced an approach with an appropriate lexicon to be used for the ultrasound report, revised and published in 2017.^5^ Similarly, the European Thyroid Association (ETA) developed a risk stratification system for thyroid nodules with a practical image guide, published in September 2017.^6^ These ultrasound‐based risk stratification systems aim to select nodules that warrant cytological diagnosis and to reduce unnecessary and not risk‐free surgery. However, there is no consensus on which TI‐RADS scoring system is the best one. Many studies demonstrated that both guidelines provide effective stratification of malignancy risk for the diagnosis of thyroid nodules using cytology as a reference.^7^, ^8^, ^9^ However, few studies employed histology as the reference standard to evaluate these ultrasound scoring systems performances.^10^, ^11^

This study aimed to evaluate and compare the accuracy and reliability of EU TI‐RADS 2017 and ACR TI‐RADS 2017 scoring systems in predicting thyroid malignancy using histology as a reference standard.

## 2 METHODS

This was a retrospective study including 99 benign and 99 malignant nodules in 156 patients who had undergone surgery for thyroid nodules in the Oto‐rhino‐laryngology department of La Rabta university hospital, Tunis, Tunisia between 2016 and 2020. Patients were included in this study if they had a complete medical file with a physical examination, a thyroid‐stimulating hormone (TSH) measurement, a detailed cervical ultrasound report with the morphological criteria necessary to classify nodules according to EU TI‐RADS and ACR TI‐RADS scoring systems, the surgical report, and the final histology result. Patients aged less than 18 years were not included.

Age, gender, indication for surgery, TSH level, ultrasound data and histopathology report were collected.

Ultrasound examinations were performed in the department of radiology of La Rabta hospital and nodules were initially classified according to the ACR TI‐RADS scoring system. We applied then the EU TI‐RADS system. This latest version of EU TI‐RADS consists of five categories, each one is scored in correspondence to features from ultrasound examination: EU TI‐RADS 1 refers to the absence of thyroid nodule and EU TI‐RADS 5 involves nodules presenting at least one of the following high‐risk signs of malignancy: nonoval shape, irregular margins, microcalcifications and marked hypoechogenicity. The other three categories correspond to an increased risk of malignancy.^6^ The ACR TI‐RADS system is based on the assessment of different US features of thyroid nodules: composition, echogenicity, shape, margin and echogenic foci. Each of these features is associated with a score ranging from 0 to 3 points. The sum of the assigned points defines the risk of malignancy according to 5 grades, with each grade corresponding to benign, not suspicious, mildly suspicious, moderately suspicious and highly suspicious for malignancy.^5^

### 2.1 Statistical analysis

Statistical analysis was performed using the SPSS software package version 22. Continuous data were expressed as mean ± standard deviation or as ranges. Categorical data were expressed using frequencies and percentages. The student's *t*‐test was used to compare continuous variables and the chi‐square test or the Fisher exact test to compare categorical variables. Ultrasound criteria predictive of malignancy were determined by calculating odd ratios (OR).

To assess the diagnostic performance of both EU TI‐RADS and ACR‐TIRADS, receiver operating characteristic (ROC) curves were constructed and the area under the curve (AUC) was calculated. The corresponding sensitivity, specificity, positive predictive value and negative predictive value were calculated. Agreement between the two systems was measured by Cohen's kappa coefficient. *p* values<0.05 were considered to indicate statistical significance.

## 3 RESULTS

A total of 198 nodules in 156 patients (127 women and 29 men) were included in this study. All of the patients underwent surgery in the ORL department of our hospital. The youngest patient was 18 years old and the oldest was 80, with a mean age of 47.9 ± 13.9 years. Preoperative TSH was normal in most patients and suppressed in 13 patients. The number of nodules per patient was one nodule in 121 patients, two nodules in 27 patients and three nodules or more in 8 patients. The indications of thyroid surgery were compression symptoms (7%), ultrasound high‐risk lesion (64%) or fine needle aspiration cytology class 5 or 6 according to the Bethesda classification (29%). Initial surgery was a total thyroidectomy in 76.3% of cases and a thyroid lobectomy in 23.7% of cases. Out of the 156 patients, 89 patients had at least one malignant nodule, and 67 patients had one or more exclusively benign nodules. Adenomatous hyperplasia was noted in all benign nodules associated with lesions of thyroiditis, which comprised 30% of cases. Among the malignant nodules, there were 86 papillary carcinomas, five follicular carcinomas, four medullary carcinomas, two oncocytic carcinomas and two undifferentiated carcinomas. Malignant nodules were significantly smaller than benign nodules (23.7 ± 17.2 mm vs. 29.8 ± 13.1 mm; *p* = .005). Compared with benign nodules, the malignant nodules were significantly more likely to be solid or mostly solid, to have a hypoechogenicity or marked hypoechogenicity, to be lobulated or to have irregular margins, to be taller‐than‐wide shaped, to have microcalcifications and to be associated with suspect lymph nodes (Table 1). However, on multivariate logistic regression analysis, none of these ultrasound signs was independently associated with the risk of malignancy.

**TABLE 1:** Ultrasound features in malignant and benign nodules (univariate analysis).

		Malignant (*n* = 99)	Benign (*n* = 99)	*p*‐value	OR	95% CI
Nodule size (mm)		23.5 ± 17.2	29.9 ± 13.1	**.004**	–	
Composition(%)	Exclusively Solid	86	44	**<10** ^**−3**^	7.81	3.90–15.57
	Solido‐cystic	14	46	**<10** ^**−3**^	0.19	0.10–0.39
	Spongiform	0	8	**.003**	0.48	0.41–0.55
	Cystic	0	2	.123	0.49	0.42–0.56
Echogenicity (%)	Hypoechoic	83	23	**<10** ^**−3**^	16.42	8.10–33.27
	Markedly hypoechoic	21	2	**<10** ^**−3**^	12.49	2.84–54.93
	Moderately hypoechoic	62	21	**<10** ^**−3**^	6.20	3.27–11.72
	Hyperechoic	7	18	**.019**	0.35	0.13–0.88
	Isoechoic	10	59	**<10** ^**−3**^	0.07	0.03–0.16
Irregular margins (%)		36	7	**<10** ^**−3**^	7.47	3.13–17.83
Shape: Taller‐than‐wide (%)		14	4	**.02**	3.58	1.12–11.41
Echogenic foci (%)	Macrocalcification	12	5	.063	2.59	0.9–7.65
	Microcalcification	22	9	**.006**	3.02	1.31–6.91
	Peripheral calcification	3	3	–	1	–
Suspicious lymphadenopathy (%)		16	5	**.006**	3.89	1.37–11

*Note*: The difference is statistically significant in bold.

Diagnostic performance of ultrasound signs in distinguishing benign from malignant nodules is detailed in Table 2. While solid composition had the best sensitivity, marked hypoechogenicity and taller‐than‐wide shape were the most specific ultrasound signs in estimating thyroid malignancy.

**TABLE 2:** Diagnostic performances of ultrasound criteria predictive of malignancy.

	Sensitivity (%)	Specificity (%)	Positive predictive value (%)	Negative predictive value (%)
Exclusively solid	86	56	66	80
Moderately hypoechoic	62	80	75	68
Marked hypoechoic	21	98	91	55
Irregular margins	36	93	84	59
Taller‐than‐wide	14	96	78	53
Microcalcifications	22	92	73	54
Suspicious lymph nodes	16	95	76	53

Table 3 represents the comparison of malignancy risk according to each category in both TI‐RADS classifications using histology as a reference standard. The two systems proposed an estimated risk of malignancy in each category. Most of them were not well matched within the range of the theoric malignancy risk. The malignancy rates tended to increase along with the higher risk categories.

**TABLE 3:** Malignant rates in the different categories of EU‐ and ACR‐TIRADS using histology as a reference standard.

Thyroid nodules classifications		Malignant nodules (*n* = 99)	Benign nodules (*n* = 99)	*p*‐value	OR	95% CI	Calculated malignancy risk (%)	Reported malignancy risk (%)
EU TI‐RADS (%)	1	0	0	–	–		0	0
	2	0	9	**.002**	0.47	0.41–0.55	0	0
	3	11	60	**<10** ^**−3**^	0.08	0.03–0.17	15.5	2–4
	4	29	13	**.004**	2.73	1.32–5.64	69	6–17
	5	60	18	**<10** ^**−3**^	6.83	3.57–13.06	76.9	26–87
	4–5	89	31	**<10** ^**−3**^	17.8	8.35–37.94	–	–
ACR TI‐RADS (%)	1	0	3	.123	0.49	0.42–0.56	0	0
	2	3	37	**<10** ^**−3**^	0.05	0.01–0.17	7.5	<2
	3	11	22	**.028**	0.43	0.20–0.96	33.3	5
	4	45	34	.074	1.58	0.89–2.81	57	5–20
	5	41	4	**<10** ^**−3**^	16.67	5.68–48.94	91.9	> 20
	4–5	86	38	**<10** ^**−3**^	10.02	5–20.06	‐	‐

*Note*: The difference is statistically significant in bold.

Table 4 represents the sensitivity, specificity, positive predictive value and the negative predictive value of the EU TI‐RADS and the ACR TI‐RADS classifications.

**TABLE 4:** Sensitivity, specificity, positive predictive value and negative predictive value for EU and ACR TI‐RADS scoring systems for predicting malignancy risk of thyroid nodules.

	SEN (%)	SPE (%)	PPV (%)	NPV (%)
EU TI‐RADS 5	60	82	77	67
ACR TI‐RADS 5	41	96	91	62
*The p‐*value for category 5	**.007**	**.002**	**.007**	NS.
EU TI‐RADS 4/5	89	69	74	86
ACR TI‐RADS 4/5	86	62	69	82
*The p*‐value for category 4/5	NS	NS	NS	NS

*Note*: The difference is statistically significant in bold.

Abbreviations: NPV, negative predictive value; NS, nonsignificant; PPV, positive predictive value; SEN, sensitivity, SPE, specificity.

**FIGURE 1:**
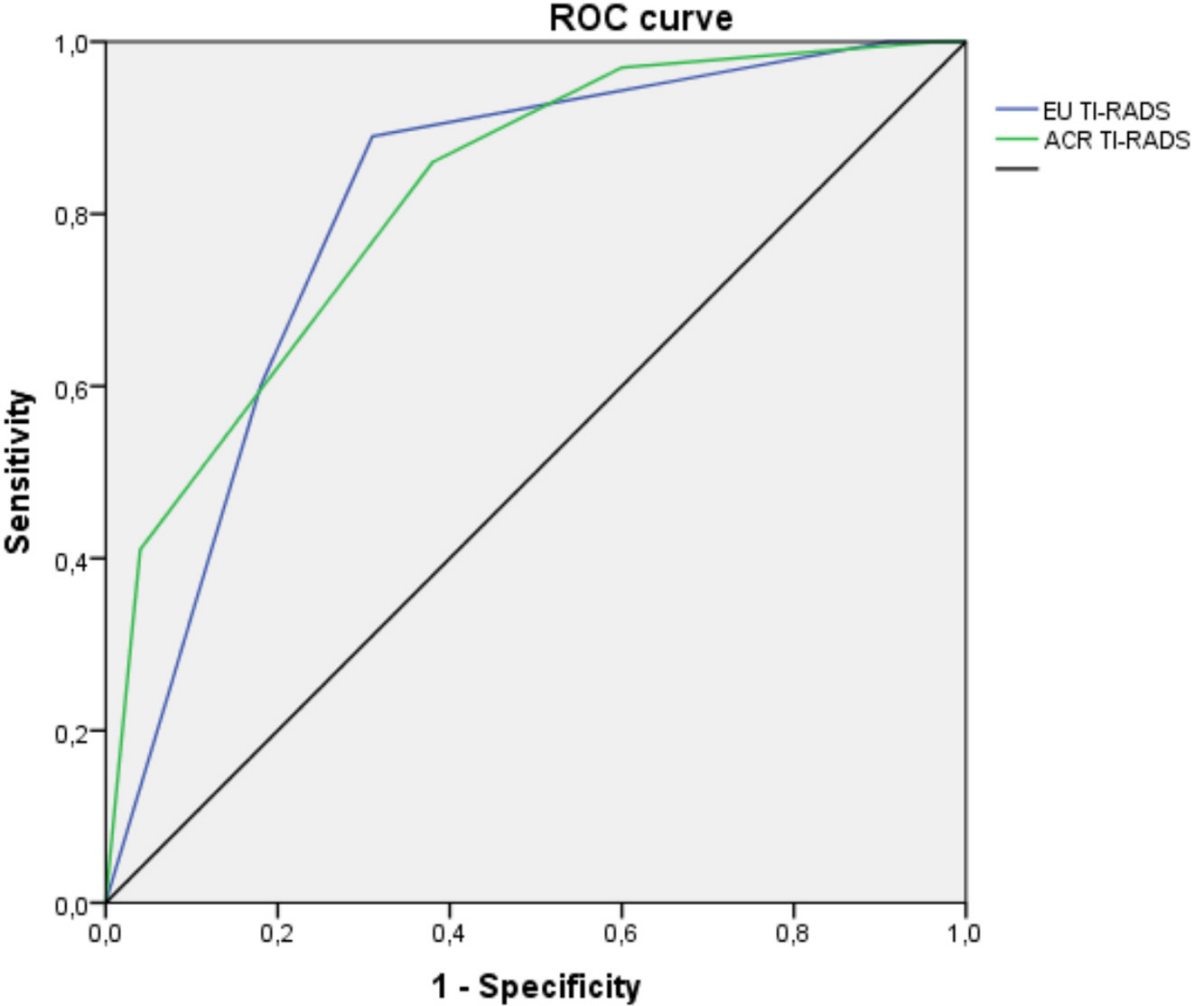
Receiver operating characteristic (ROC) curves for both TI‐RADS scoring systems for predicting malignancy risk.

Figure 1 represents the ROC curve analysis for predicting malignancy according to the ACR TI‐RADS and EU TI‐RADS scoring systems. The area under the ROC curve was 0.81 (95% CI = 0.75–0.87, *p* < 10^−3^) for EU TI‐RADS and 0.82 (95% CI = 0.76–0.87, *p* < 10^−3^) for ACR TI‐RADS.

In the assessment of concordance between EU TI‐RADS and ACR TI‐RADS (Table 5), 40.8% of EU TI‐RADS category 3 nodules, 90.5% of category 4 and 57.9% of category 5 nodules corresponded to ACR TI‐RADS categories 3, 4 and 5, respectively. The mean concordance rate of all categories was 59%. The mean Cohen's kappa coefficient was 0.46 indicating a moderate agreement. The best kappa value was found between both categories 4 and 5 of the classification systems (Cohen's kappa = 0.87).

**TABLE 5:** Concordance of classified categories between EU TI‐RADS and ACR TI‐RADS scoring systems.

		ACR TI‐RADS					Total
		1	2	3	4	5	
EU TI‐RADS	2	3	**6**	0	0	0	9
	3	0	34	**29**	8	0	71
	4	0	0	4	**38**	0	42
	5	0	0	0	32	**44**	76
Total		3	40	33	78	44	198

*Note*: The difference is statistically significant in bold.

## 4 DISCUSSION

Our results affirmed the good performance of both EU TI‐RADS and ACR TI‐RADS 2017 ultrasound classifications in detecting malignant thyroid nodules.

In adjunction to clinical assessment, ultrasound examination of the thyroid is a useful tool to characterize nodules and to indicate the FNA cytology, hence making the right decision for a follow‐up or surgical treatment.^12^ Reliable and reproducible ultrasound criteria have been defined in recent years to distinguish suspicious nodules from benign ones.^13^ The ultrasound features associated with a high risk of malignancy are of variable importance and none of them is sensitive enough to guide clinical decisions when considered separately. However, their association is more specific.^9^, ^10^, ^11^ Our study showed that the exclusively solid composition, hypoechogenicity, taller‐than wide shape, irregular margins and microcalcifications were significantly associated with malignancy. These findings were in line with what was previously described in many studies.^8^, ^14^, ^15^, ^16^, ^17^ The diagnostic performance of each ultrasound criteria was determined by calculating the sensitivity, specificity and positive and negative predictive values. The exclusively solid composition had the best sensitivity. Marked hypoechogenicity and taller‐than‐wide shape were the more specific ultrasound features. Our results were consistent with the findings of other studies.^18^, ^19^

As none of these ultrasound features alone is sufficient to predict malignancy, several international thyroid societies have developed ultrasound classifications that combine several features to improve their diagnostic performance for thyroid nodules. In 2009, Horvath et al.^20^ had the idea of extrapolating the concept of BI‐RADS (Breast imaging reporting and data system), widely used in breast cancer, to the ultrasound evaluation of thyroid nodules and thus proposed the TI‐RADS scoring system. In recent years, many versions have been proposed but no TI‐RADS classification has been widely accepted.^8^ Therefore, we aimed to find out the most reproducible and precise guideline among the most commonly used classification systems; EU TI‐RADS 2017 and ACR TI‐RADS 2017. For both classifications, the malignancy rate increased along with the higher risk categories. Category 5 was significantly associated with thyroid cancer with an OR of 6.83 with the EU TI‐RADS and 16.67 with the ACR TI‐RADS. Trimboli et al.^10^ also found a significant association between category 5 according to the EU TI‐RADS and thyroid cancer with an OR of 84.7.

As for the EU‐TIRADS, the risk of malignancy is consistent with that established by the ETA for categories 2 and 5. For categories 3 and 4, the prevalence of malignancy was relatively higher than the reported values but was in accordance with the results of Shi et al.^7^

Similarly for the ACR TI‐RADS, although the risks of malignancy found were significantly higher than reported values, they are close to the results of several other series.^7^, ^9^, ^21^ Since this is a retrospective analysis, patients in this study were all selected from a surgery department, which may lead to being classified at a higher risk for malignancy.

In this study, we evaluated the diagnostic performance of these two international TI‐RADS for detecting thyroid cancer. When we considered category 5 as the cut‐off value, the EU TI‐RADS classification showed a significantly higher sensitivity and the ACR TI‐RADS classification had a higher specificity. However, no significant difference was noted with either classification when both categories 4 and 5 were compared to the other categories.

Our study showed that the category‐based diagnostic performances of both classifications were closely comparable. But, the sensitivity and specificity of each system varied depending on which category was considered as the positive result (Category 5 or categories 4 and 5). These results were consistent with those of two recent meta‐analyses.^22^, ^23^ If only category 5 is considered, the EU TI‐RADS system would have a better diagnostic performance than the ACR TI‐RADS system but without a significant difference. This could be explained by the fact that the presence of one ultrasound sign highly predictive of malignancy (nonoval shape, irregular contours, marked hypoechogenicity, microcalcifications) is sufficient to classify the nodule in category 5, whereas for the ACR TI‐RADS system these ultrasound signs do not all have the same value. If the two categories 4 and 5 are considered together, the diagnostic performance of both ultrasound classification systems overlaps with a sensitivity exceeding 85%, but with a lower specificity.

Receiver operating characteristic (ROC) curves were used for the assessment of the performance of the TI‐RADS classifications. The area under the ROC curve was 0.81 for the EU TI‐RADS classification and 0.82 for the ACR TI‐RADS classification. Our results were close to those described in the literature where the area under the ROC curve varied between 0.76 and 0.91 for the EU TI‐RADS classification^7^, ^10^, ^11^ and between 0.82 and 0.88 for the ACR TI‐RADS classification.^7^, ^11^, ^24^, ^25^

In our study, the EU and ACR TI‐RADS classified the nodules in the same category in 59% of cases. The percentages of agreement between EU TI‐RADS and ACR TI‐RADS were 40.8%, 90.4% and 57.7% for categories 3, 4 and 5, respectively. In the study of Yoon et al.,^26^ these percentages were 42.3%, 41% and 49.7%. So, we can conclude that the level of agreement was average for categories 3 and 4, good for category 5 and very good for categories 4 and 5. The overall agreement measure between the two ultrasound classifications showed a kappa coefficient of 0.46 indicating a moderate level of agreement.

The strength of our study was the use of histology as a reference for the comparison of two ultrasound classifications of thyroid nodules which leads to a precise and accurate evaluation of the two risk stratification systems. However, several limitations of our work should also be addressed. Firstly, the retrospective nature of the study and the retrieval of ultrasound features from recorded reports might lead to unavoidable bias. Secondly, there may be inter‐rater differences in the evaluation of the characteristics of thyroid nodules as the ultrasound was not performed by the same radiologist. Prospective studies with ultrasound performed by the same qualified radiologist may overcome these limitations.

## 5 CONCLUSION

Both ACR TI‐RADS and EU TI‐RADS scoring systems provided effective stratification of malignancy risk for the diagnosis of thyroid nodules.

## AUTHOR CONTRIBUTIONS

**Hiba‐Allah Chatti:** Conceptualization (supporting); data curation (equal); formal analysis (equal); investigation (equal); methodology (equal); resources (equal); software (equal); validation (equal); writing – original draft (lead); writing – review and editing (supporting). **Ibtissem Oueslati:** Conceptualization (lead); data curation (equal); formal analysis (equal); investigation (equal); methodology (lead); project administration (lead); resources (equal); software (equal); supervision (lead); validation (equal); visualization (equal); writing – original draft (supporting); writing – review and editing (equal). **Aymen Azaiez:** Data curation (supporting); formal analysis (supporting); investigation (supporting); resources (supporting); validation (supporting); visualization (supporting); writing – review and editing (supporting). **Jihene Marrakchi:** Conceptualization (supporting); data curation (supporting); formal analysis (supporting); investigation (equal); methodology (supporting); resources (equal); validation (supporting); visualization (supporting); writing – review and editing (supporting). **Seif Boukriba:** Investigation (supporting); methodology (supporting); resources (supporting); validation (supporting); visualization (supporting); writing – review and editing (supporting). **Habiba Mizouni:** Data curation (supporting); investigation (supporting); resources (supporting); validation (supporting); visualization (supporting); writing – review and editing (supporting). **Slim Haouet:** Data curation (supporting); investigation (supporting); resources (supporting); validation (supporting); visualization (supporting); writing – review and editing (supporting). **Ghazi Besbes:** Data curation (supporting); investigation (supporting); resources (supporting); validation (supporting); visualization (supporting); writing – review and editing (supporting). **Meriem Yazidi:** Conceptualization (supporting); data curation (supporting); investigation (supporting); resources (supporting); validation (supporting); visualization (supporting); writing – review and editing (supporting). **Melika Chihaoui:** Conceptualization (supporting); data curation (supporting); formal analysis (supporting); investigation (supporting); methodology (supporting); resources (supporting); software (supporting); validation (equal); writing – review and editing (supporting).

## FUNDING INFORMATION

This research did not receive any specific grant from funding agencies in the public, commercial or not‐for‐profit sectors.

## CONFLICT OF INTEREST STATEMENT

The authors declare that they have no competing interests.

## ETHICS STATEMENT

The study was carried out in accordance with the ethical standards of the institutional and the national research committee and with the 1964 Helsinki declaration.

## Data Availability

The data used and analysed during the current study are available from the corresponding author on reasonable request.

## References

[1] Haugen BR , Alexander EK , Bible KC , et al. 2015 American Thyroid Association management guidelines for adult patients with thyroid nodules and differentiated thyroid cancer: the American Thyroid Association guidelines task force on thyroid nodules and differentiated thyroid cancer. Thyroid. 2016;26(1):1‐133. 26462967 10.1089/thy.2015.0020 PMC4739132

[2] Biermann M . Thyroid ultrasound classification system accurately predicts risk of malignancy in subcentimeter nodules. Clin Thyroidol. 2018;30:273‐276.

[3] Gharib H , Papini E , Garber JR , et al. American Association of Clinical Endocrinologists, American College of Endocrinology, and associazione medici endocrinologi medical guidelines for clinical practice for the diagnosis and management of thyroid nodules—2016 update. Endocr Pract. 2016;22(5):622‐639. 27167915 10.4158/EP161208.GL

[4] Farihah AG , Nurismah MI , Husyairi H , Shahrun Niza AS , Radhika S . Reliability of the ultrasound classification system of thyroid nodules in predicting malignancy. Med J Malaysia. 2018;73(1):9‐15. 29531197

[5] Tessler FN , Middleton WD , Grant EG , et al. ACR thyroid imaging, reporting and data system (TI‐RADS): white paper of the ACR TI‐RADS Committee. J Am Coll Radiol. 2017;14(5):587‐595. 28372962 10.1016/j.jacr.2017.01.046

[6] Russ G , Bonnema SJ , Erdogan MF , Durante C , Ngu R , Leenhardt L . European thyroid association guidelines for ultrasound malignancy risk stratification of thyroid nodules in adults: the EU‐TIRADS. Eur Thyroid J. 2017;6(5):225‐237. 29167761 10.1159/000478927 PMC5652895

[7] Shi YX , Chen L , Liu YC , et al. Differences among the thyroid imaging reporting and data system proposed by Korean, the American College of Radiology and the European thyroid association in the diagnostic performance of thyroid nodules. Transl Cancer Res. 2020;9(8):4958‐4967. 35117857 10.21037/tcr-19-2870 PMC8798608

[8] Shen Y , Liu M , He J , et al. Comparison of different risk‐stratification Systems for the Diagnosis of benign and malignant thyroid nodules. Front Oncol. 2019;9:378. 31139568 10.3389/fonc.2019.00378 PMC6527759

[9] Xu T , Wu Y , Wu RX , et al. Validation and comparison of three newly‐released thyroid imaging reporting and data systems for cancer risk determination. Endocrine. 2019;64(2):299‐307. 30474824 10.1007/s12020-018-1817-8

[10] Trimboli P , Ngu R , Royer B , et al. A multicentre validation study for the EU‐TIRADS using histological diagnosis as a gold standard. Clin Endocrinol. 2019;91(2):340‐347. 10.1111/cen.13997 31002419

[11] Magri F , Chytiris S , Croce L , et al. Performance of the ACR TI‐RADS and EU TI‐RADS scoring systems in the diagnostic work‐up of thyroid nodules in a real‐life series using histology as reference standard. Eur J Endocrinol. 2020;183(5):521‐528. 32841935 10.1530/EJE-20-0682

[12] Koc AM , Adıbelli ZH , Erkul Z , Sahin Y , Dilek I . Comparison of diagnostic accuracy of ACR‐TIRADS, American Thyroid Association (ATA), and EU‐TIRADS guidelines in detecting thyroid malignancy. Eur J Radiol. 2020;133:109390. 33181485 10.1016/j.ejrad.2020.109390

[13] Russ G , Bigorgne C , Royer B , Rouxel A , Bienvenu‐Perrard M . The thyroid imaging reporting and data system (TIRADS) for ultrasound of the thyroid. J Radiol. 2011;92:701‐713. 21819912 10.1016/j.jradio.2011.03.022

[14] Witczak J , Taylor P , Chai J , et al. Predicting malignancy in thyroid nodules: feasibility of a predictive model integrating clinical, biochemical, and ultrasound characteristics. Thyroid Res. 2016;9:4. 27313663 10.1186/s13044-016-0033-y PMC4910190

[15] Canete EJ , Sison‐Pena CM , Jimeno CA . Clinicopathological, biochemical, and sonographic features of thyroid nodule predictive of malignancy among adult Filipino patients in a tertiary hospital in The Philippines. Endocrinol Metab (Seoul). 2014;29(4):489‐497. 25325270 10.3803/EnM.2014.29.4.489 PMC4285043

[16] Liu J , Zheng D , Li Q , et al. A predictive model of thyroid malignancy using clinical, biochemical and sonographic parameters for patients in a multi‐center setting. BMC Endocr Disord. 2018;18(1):17. 29514621 10.1186/s12902-018-0241-7 PMC5842594

[17] Arpana POB , Gurung G , Pradhan S . Ultrasound findings in thyroid nodules: a radio‐cytopathologic correlation. J Med Ultrasound. 2018;26(2):90‐93. 30065526 10.4103/JMU.JMU_7_17 PMC6029197

[18] Popoveniuc G , Jonklaas J . Thyroid nodules. Med Clin North Am. 2012;96:329‐349. 22443979 10.1016/j.mcna.2012.02.002 PMC3575959

[19] Hoang JK , Lee WK , Lee M , Johnson D , Farrell S . US features of thyroid malignancy: pearls and pitfalls. Radiographics. 2007;27(3):847‐860. 17495296 10.1148/rg.273065038

[20] Horvath E , Majlis S , Rossi R , et al. An ultrasonogram reporting system for thyroid nodules stratifying cancer risk for clinical management. J Clin Endocrinol Metab. 2009;94(5):1748‐1751. 19276237 10.1210/jc.2008-1724

[21] Ahmadi S , Oyekunle T , Scheri R , et al. A direct comparison of the ATA and TI‐RADS ultrasound scoring systems. Endocr Pract. 2019;25(5):413‐422. 30720343 10.4158/EP-2018-0369

[22] Kim PH , Suh CH , Baek JH , Chung SR , Choi YJ , Lee JH . Diagnostic performance of four ultrasound risk stratification systems: a systematic review and meta‐analysis. Thyroid. 2020;30(8):1159‐1168. 32303153 10.1089/thy.2019.0812

[23] Kim DH , Chung SR , Choi SH , Kim KW . Accuracy of thyroid imaging reporting and data system category 4 or 5 for diagnosing malignancy: a systematic review and meta‐analysis. Eur Radiol. 2020;30(10):5611‐5624. 32356157 10.1007/s00330-020-06875-w

[24] Floridi C , Cellina M , Buccimazza G , et al. Ultrasound imaging classifications of thyroid nodules for malignancy risk stratification and clinical management: state of the art. Gland Surg. 2019;8(3):S233‐S244. 31559190 10.21037/gs.2019.07.01 PMC6755949

[25] Ha SM , Ahn HS , Baek JH , et al. Validation of three scoring risk‐stratification models for thyroid nodules. Thyroid. 2017;27(12):1550‐1557. 29108488 10.1089/thy.2017.0363

[26] Yoon SJ , Na DG , Gwon HY , et al. Similarities and differences between thyroid imaging reporting and data systems. AJR Am J Roentgenol. 2019;213(2):W76‐W84. 30917027 10.2214/AJR.18.20510

